# Supplementary figures and images for: Titan cells formation in Cryptococcus neoformans is finely tuned by environmental conditions and modulated by positive and negative genetic regulators
Source: PLoS Pathog. 2018 May 18;14(5):e1006982. doi: 10.1371/journal.ppat.1006982 (PMC5959062; doi:10.1371/journal.ppat.1006982)

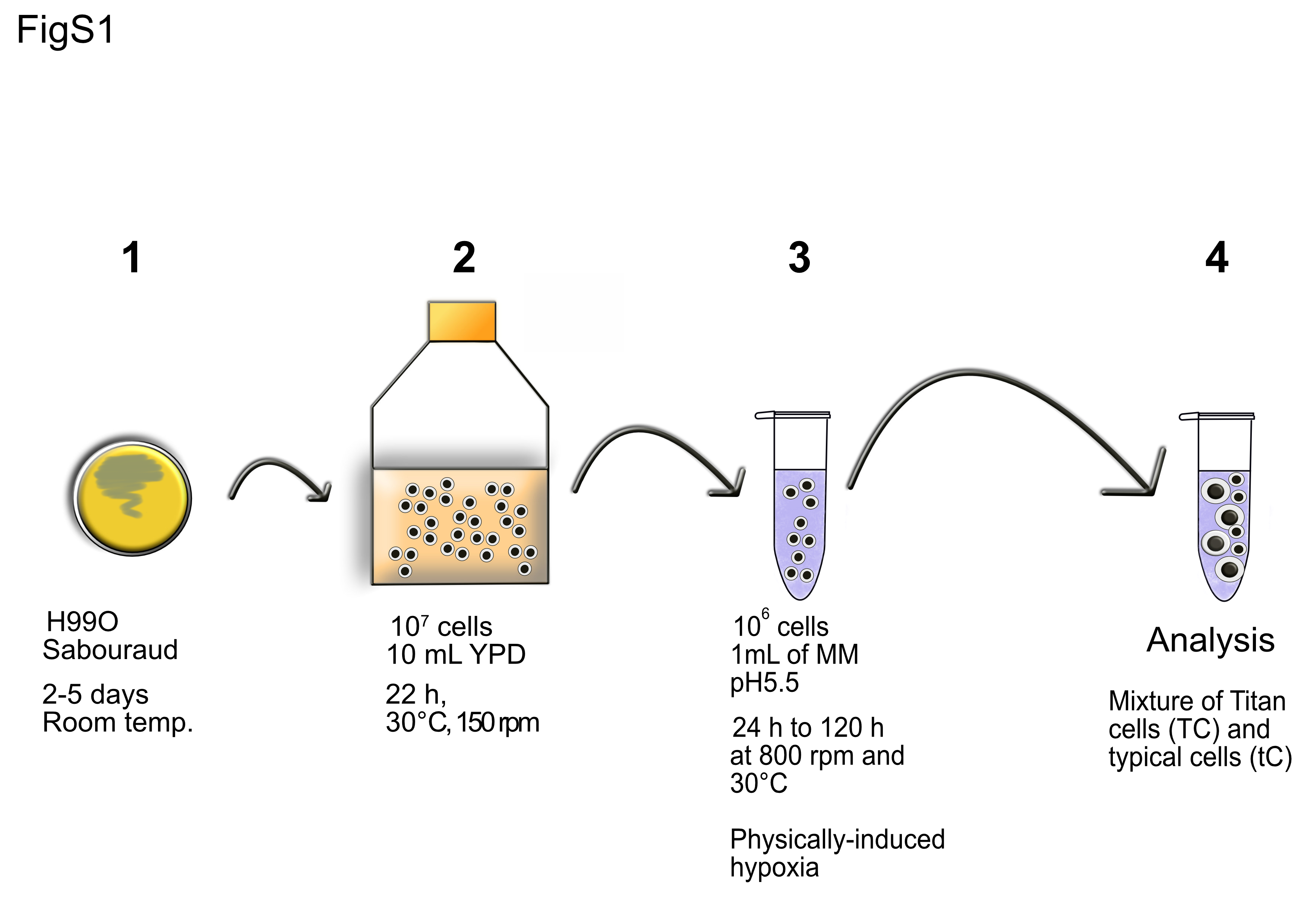

Supplement: S1 Fig — The protocol followed four steps: (1) C. neoformans H99O from a frozen stock culture at -80°C was cultured on Sabouraud agar for 2–5 d; (2) Approximately 107 yeasts were then suspended in 10mL of liquid Yeast Peptone dextrose (YPD) and incubated under agitation (150 rpm) at 30°C for 22 h (stationary phase); (3) 1 mL of the culture was then washed twice in minimal medium (MM), then 106 yeasts were resuspended in 1mL of minimal medium (MM) pH5.5, in a 1.5 mL Eppendorf tube and incubated at 800 rpm for up to 120 h using an Eppendorf thermomixer; (4) A mixture of typical cells and of titan cells was ready for analysis. (TIFF) [file ppat.1006982.s001.tiff]

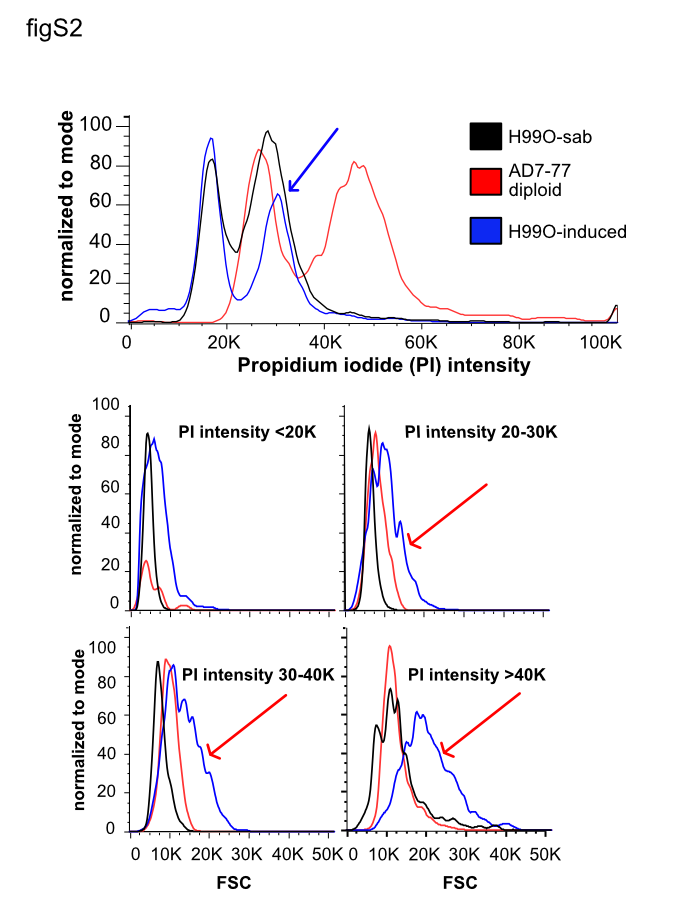

Supplement: S2 Fig — DNA content was analyzed after propidium iodide (PI) staining of yeast cells obtained at the end of our protocol (H99O induced), in a control haploid strain (H99O cultured in Sabouraud agar, H99O-sab) and in a control diploid strain (AD7-77 cultured in Sabouraud agar). Part of the population of H99O-induced had a higher PI (blue arrow) fluorescence intensity than the haploid control (upper panel). Gating on the PI intensity showed that the increase in the PI fluorescence intensity from <20K to >40K corresponded to increase in cell size (FSC) (red arrows) compared to the diploid (AD7-77) and haploid (H99O Sab) control (lower panel). (TIFF) [file ppat.1006982.s002.tiff]

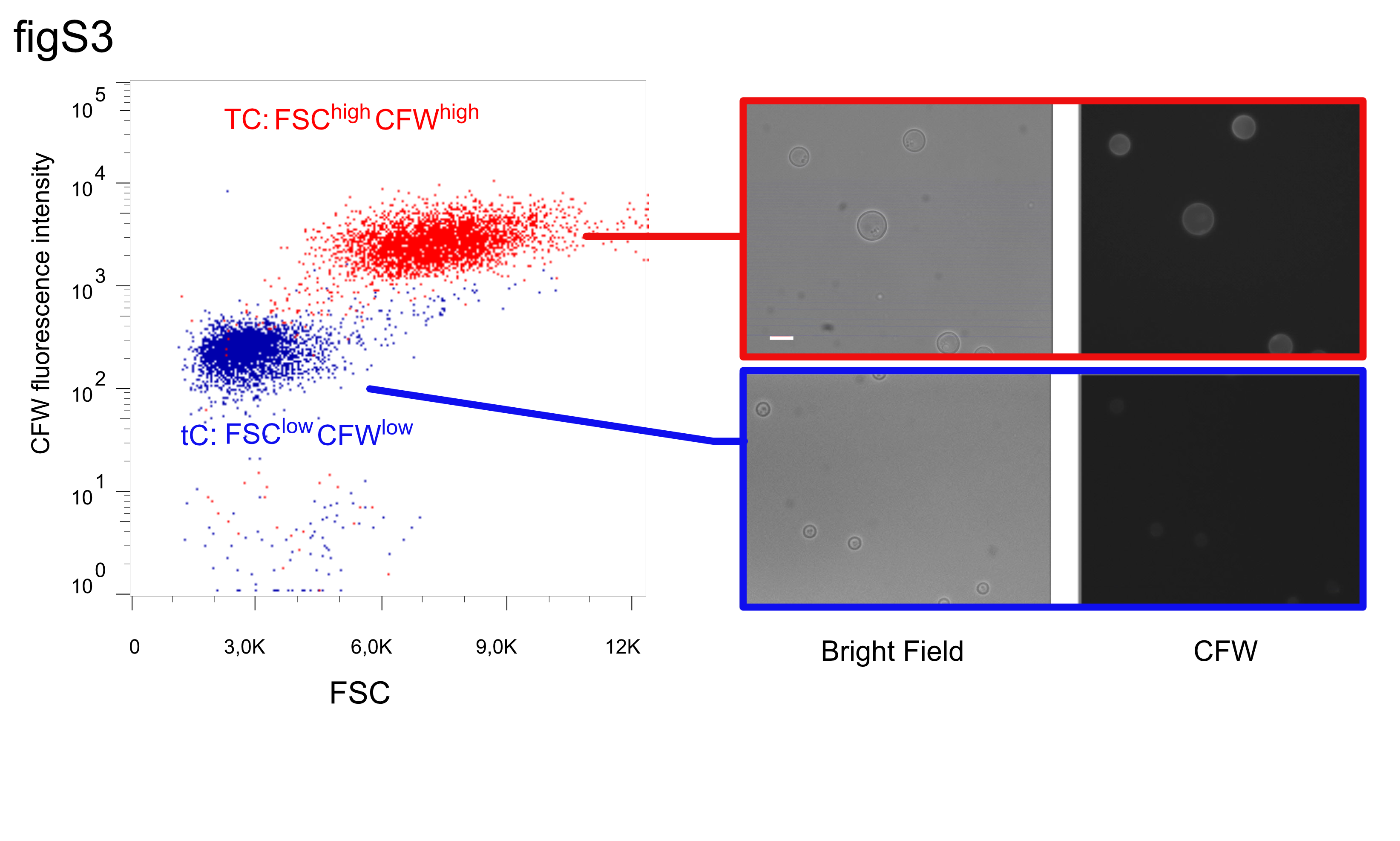

Supplement: S3 Fig — Cells obtained using our in vitro protocol were stained with CFW and sorted by flow cytometry according to size (FSC) and CFW fluorescence intensity (left panel). Sorted yeasts were observed using bright field and fluorescence microscopy (right panel) (bar = 10μm). Typical cells (tC) were FSClow/CFWlow. (TIFF) [file ppat.1006982.s003.tiff]

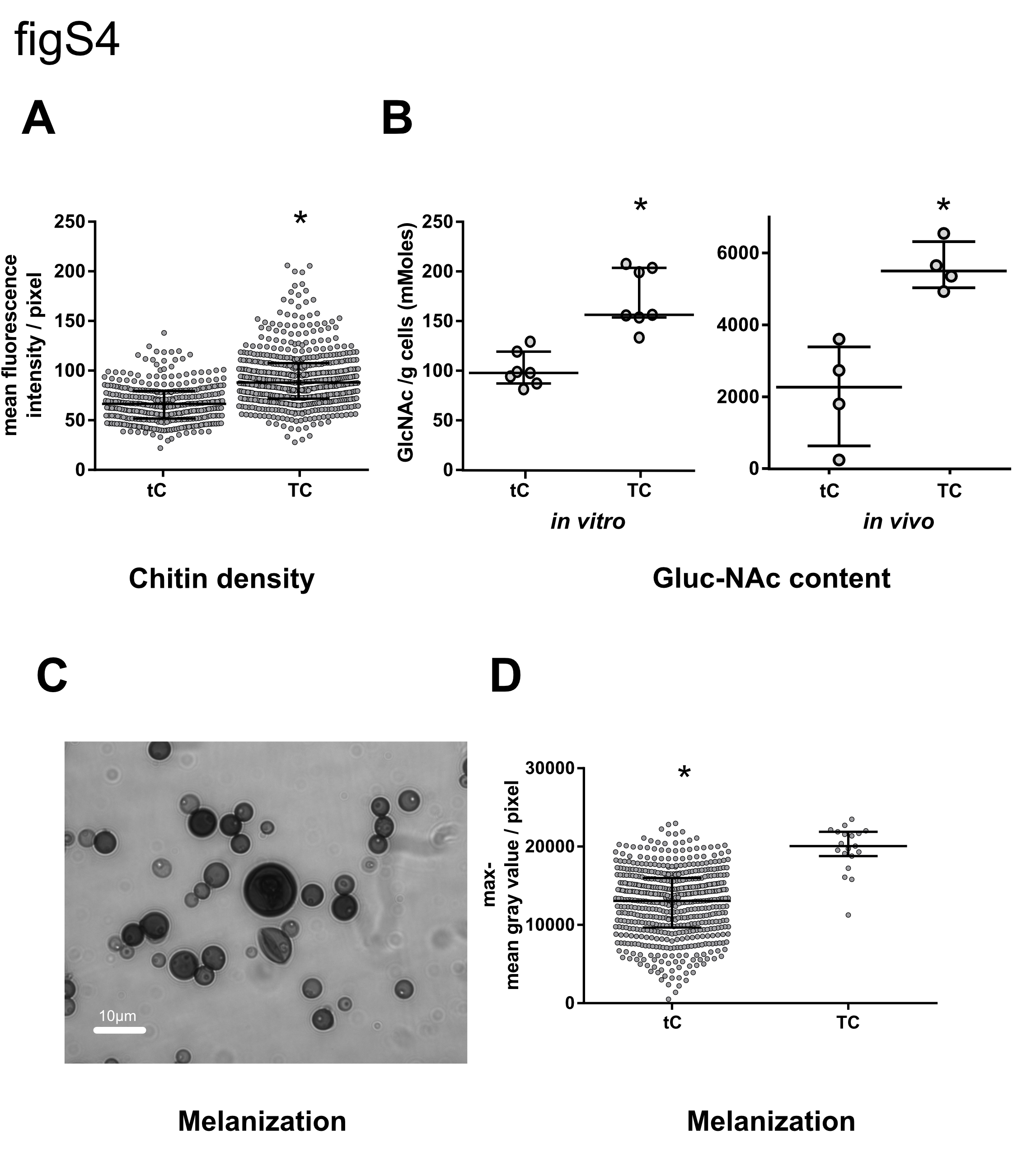

Supplement: S4 Fig — (A) Chitin was denser in titan cells (TC) than in typical cells (tC) according to CFW fluorescence intensity/pixel/cell measured by Icy software after CFW staining (0.01 μg/mL) at step 4 of the protocol (*p<0.0001). Dots represent individual cells, and boxes median and IQR for 400 cells each (*p<0.001, pooled measurements from 3 independent experiments). (B) N-acetylglucosamine (GlcNAc), the monomer component of chitin, was increased in titan cells (TC) compared to typical cells (tC) in vitro (left panel) and in vivo (right panel) as measured by a biochemical method after gamma-irradiation of the yeasts to remove the capsule, allowing a better separation of titan cells and typical cells. Each dot represents result from independent experiments (n = 7). Results are presented as median and IQR (p<0.001). (C) Comparing the blackness of the cell body of titan cells (TC) and typical cells (tC) upon melanization conditions showed that titan cells contained more melanin than typical cells. (Bar = 10μm). (D) Melanization was more important in titan cells (TC) than typical cells (tC) (*p<0.0001) based on the calculation of the max—mean grey value/pixel of each melanin ghost measured (n = 19 for titan cells and n = 531 for typical cells) using the ImageJ in Icy software. Each dot represents an individual cells and boxes median and IQR. (TIFF) [file ppat.1006982.s004.tiff]

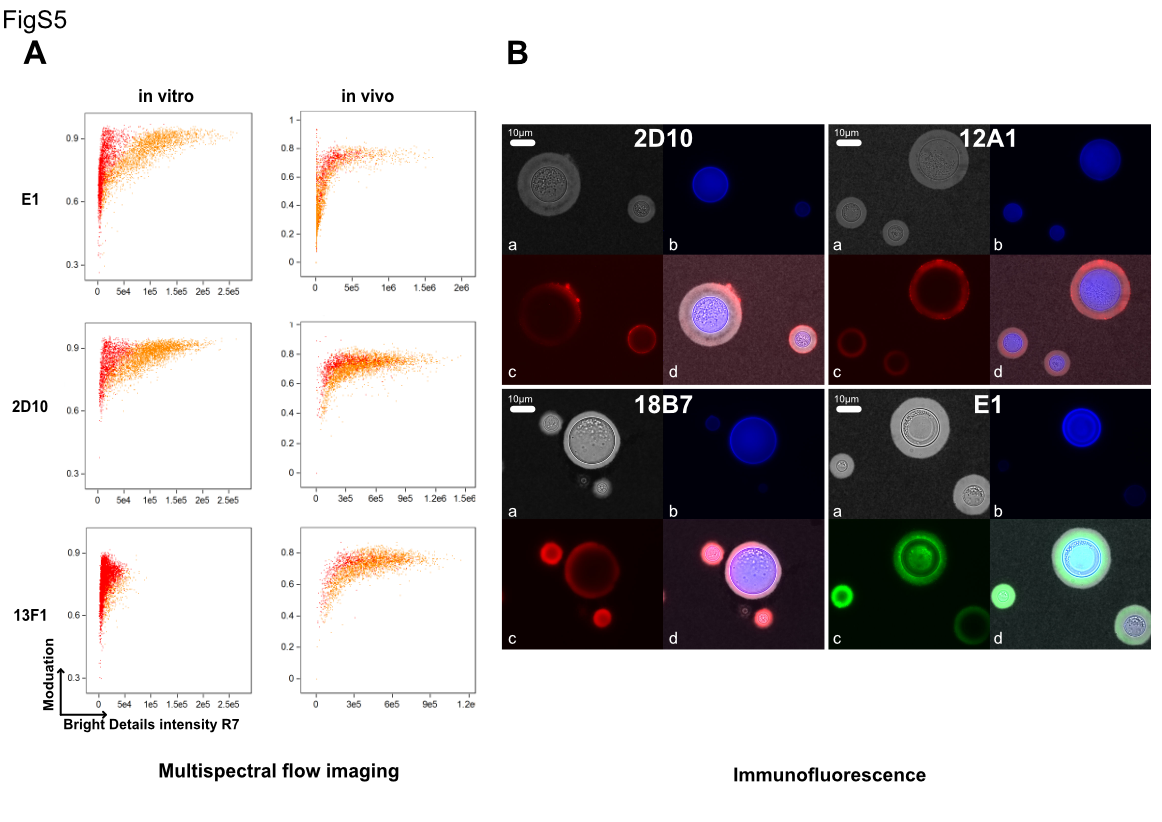

Supplement: S5 Fig — (A) Using multispectral flow cytometry and capsule staining using anticapsular monoclonal antibodies (mAb), we discriminated the distribution of titan cells and typical cells with almost no overlap between both population with 2D10 mAb in vitro and in vivo, based on the algorithm modulation and Bright details intensity R7. Overlap in the staining characteristics of titan cells and typical cells were observed for E1 (IgG1) and 13F1 (IgM) antibodies. (B) Immunofluorescence staining with the anti-capsular mAbs 2D10, 12A1, 18B7, and E1 does not uncover major differences in capsular structures between titan cells (white arrows) and typical cells (black arrows). Each panel correspond to the same cells observed after staining with (a) India ink; (b) calcofluor white; (c) one of the Mabs; (d) merge of c and d. (bar = 10μm). (TIFF) [file ppat.1006982.s005.tiff]

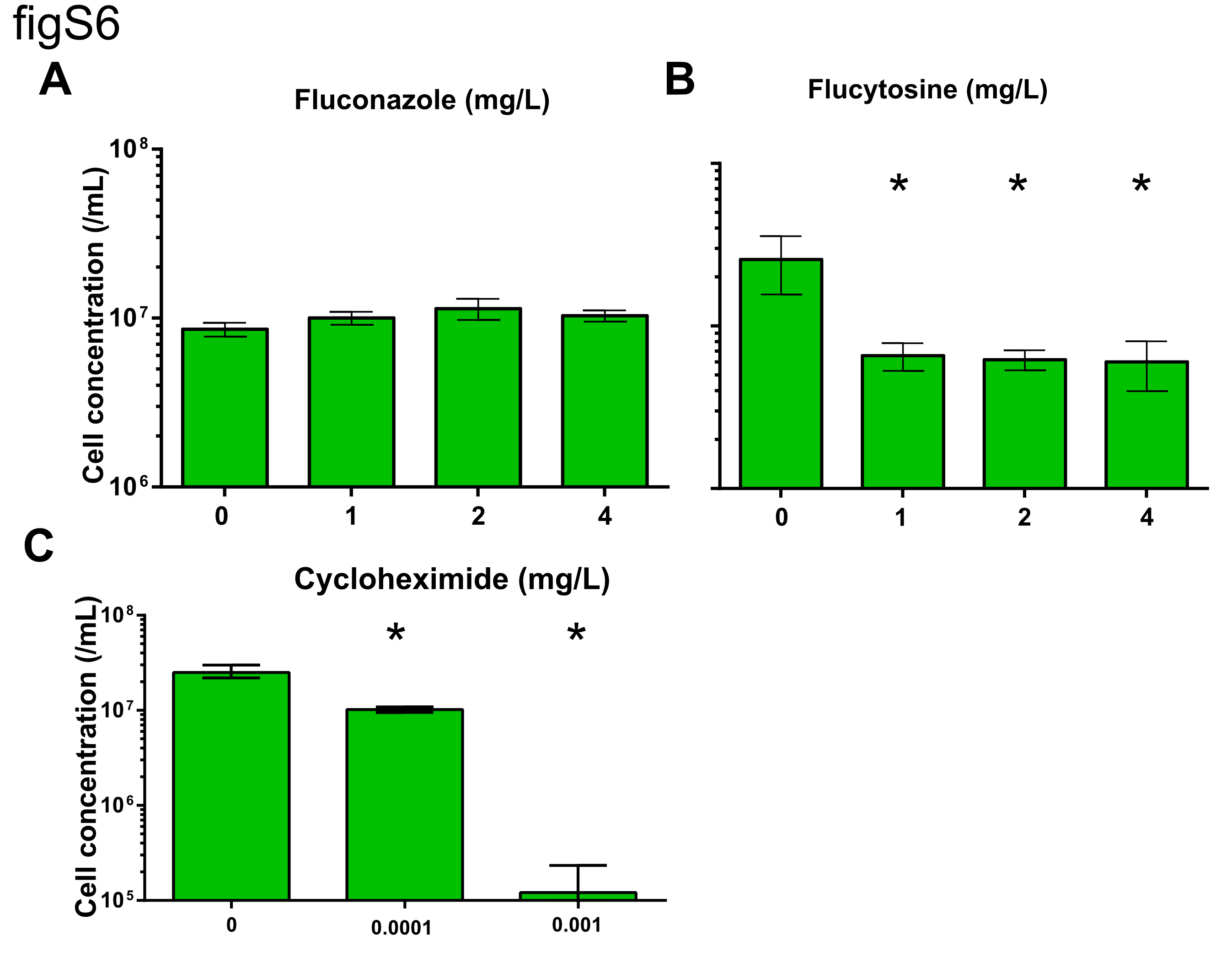

Supplement: S6 Fig — 106 cell/mL were inoculated at step 1 of our protocol, then cell growth was evaluated by enumerating cell concentration obtained at step 4 of our protocol using Guava flow cytometer for cell counting. (A) Compared to control, fluconazole did not modify cell growth in MM whereas (B) flucytosine (5FC) reduced it, when used at concentration near the minimum inhibitory concentration (MIC) for 5 d (*p<0.0001 compared to unexposed control). The fluconazole and flucytosine MICs for H99O were 8 mg/L and 4 mg/L, respectively. Experiments were done in triplicates (bars represent mean ± SD). (C) Cycloheximide also reduced cell growth at 0.0001 and inhibit cell growth at 0.001 mg/mL (*p<0.0001 compared to unexposed control). (TIFF) [file ppat.1006982.s006.tiff]

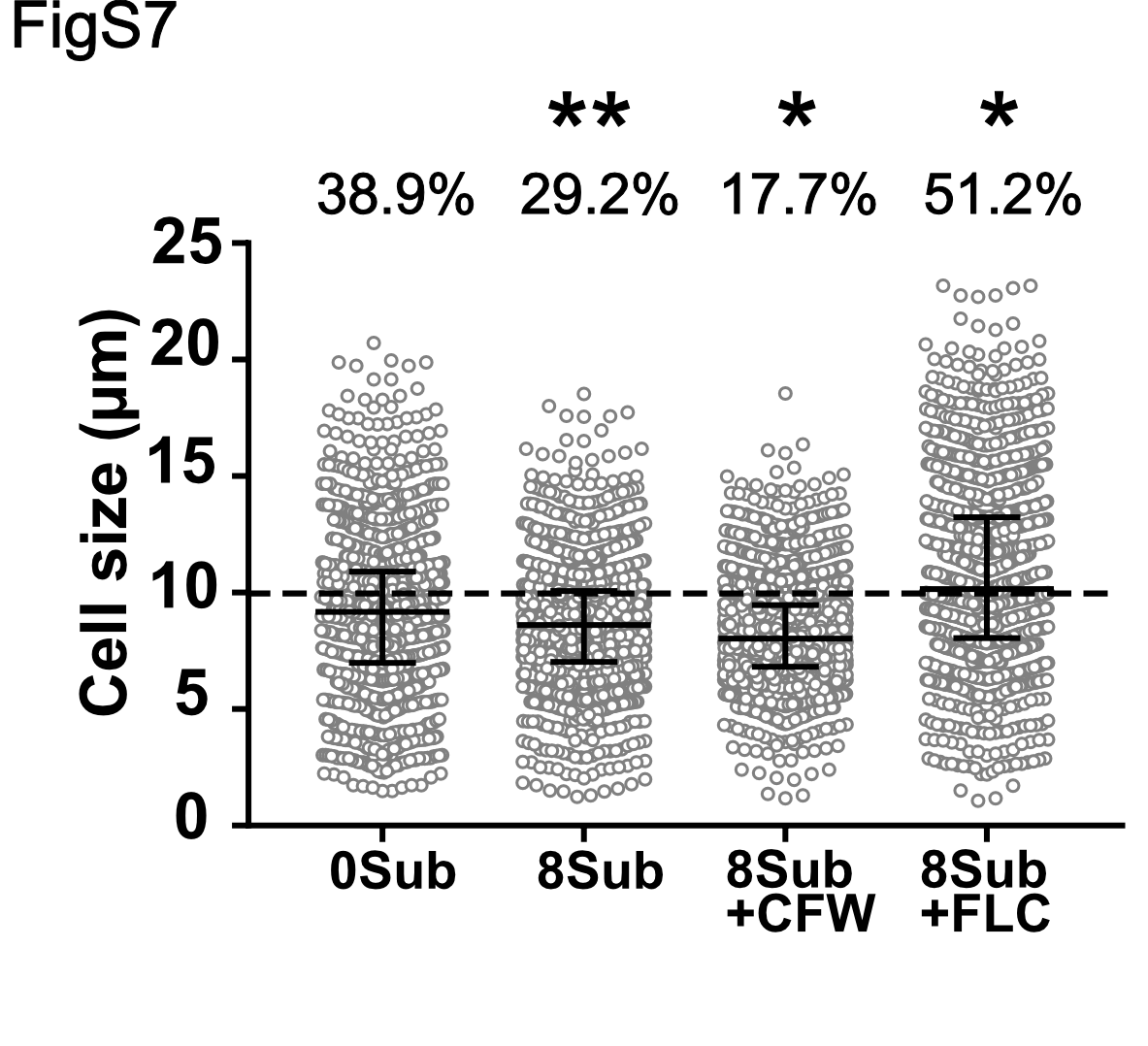

Supplement: S7 Fig — Step 1 was modified by sub-culturing H99O 8 times over one month on Sabouraud agar alone (Sub8), or supplemented with 20mg/L CFW (Sub8+CFW) or with 32mg/L fluconazole (Sub8+FLC). Compared to initial culture (0Sub), 8 sub-cultures (8Sub) decreased significantly the cell size (** p<0.0001, vs 0Sub control). In addition, iterative subcultures on CFW and FLC decreased and increased significantly the cell size compared to the 8Sub control, respectively. Median and IQR are shown in black for each condition (* p<0.0001 vs 8Sub control). The numbers above each condition represent the proportion of titan cells observed. The experiments were performed in triplicate and pooled (mean cell counted ± SD = 2455±913). (TIFF) [file ppat.1006982.s007.tiff]

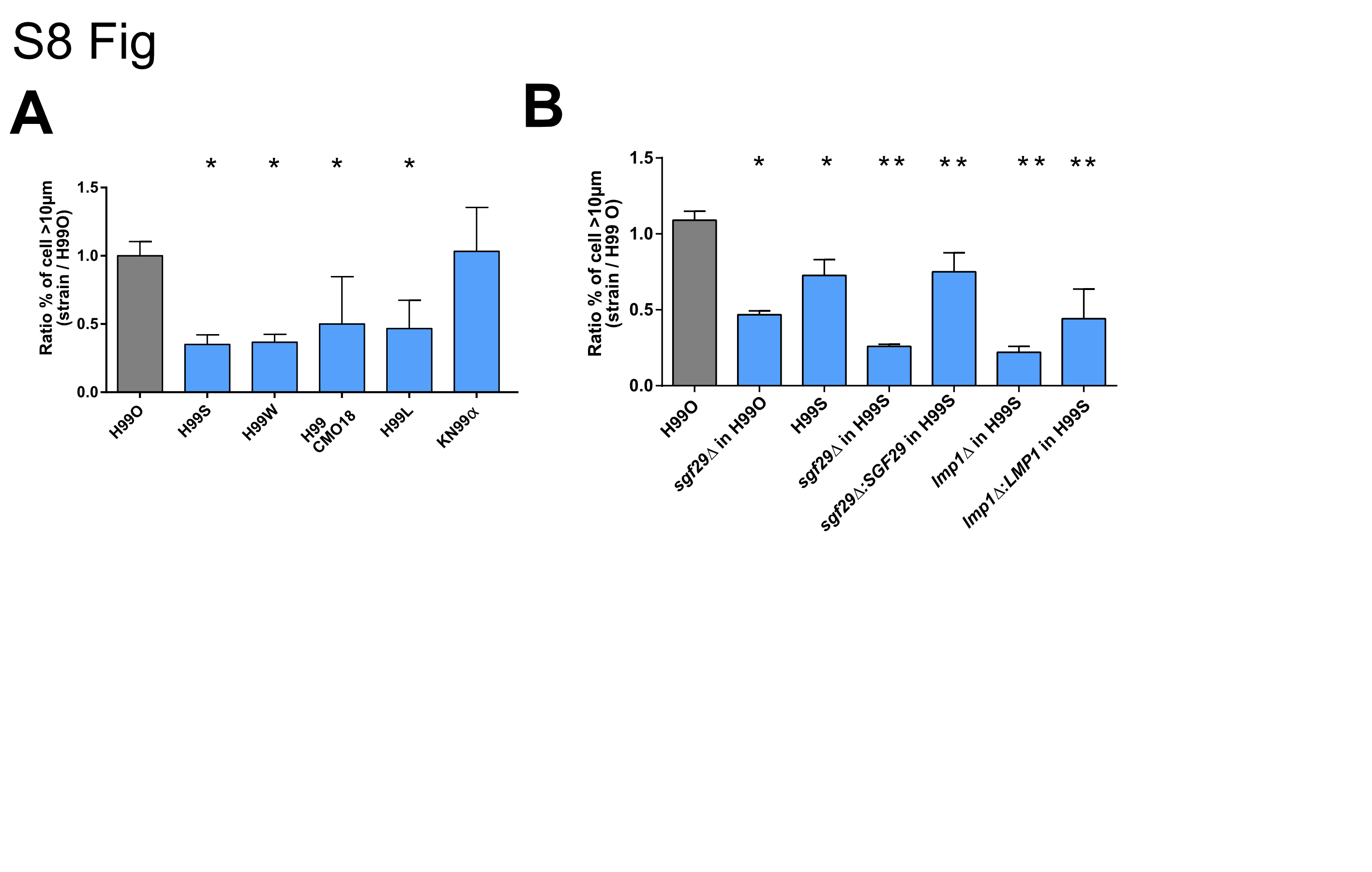

Supplement: S8 Fig — (A) Strains from the H99 lineage harbored variable abilities to generate titan cells with H99 being the main producer in in vitro and H99O and KN99α in vivo ((B)) compared to the other H99strains (S, L, W, CMO18). (C) The sgf29Δ and lmp1Δ mutant strains show a decrease in titan cells generation in various H99 backgrounds in vitro and (D) in vivo compared to H99O. (E) Rim101 and PKA pathway is required for titan cells generation in vitro in H99. Each experiment was done in triplicates. Results are presented as stacked bar of the proportion of titan cells (titan cells) and regulars cells (typical cells), * p<0.0001 vs control H99O. (TIFF) [file ppat.1006982.s008.tiff]

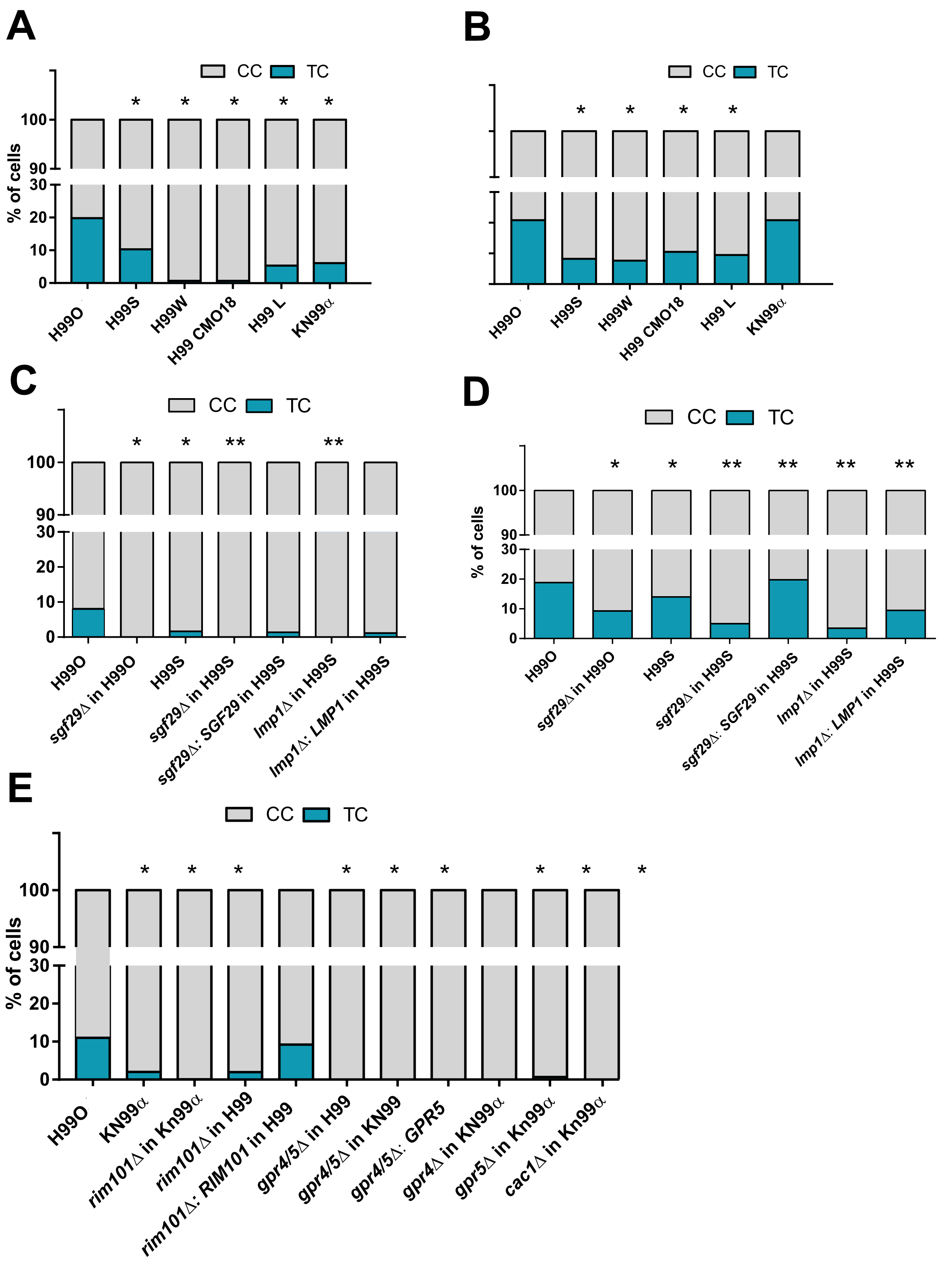

Supplement: S9 Fig — (A) The different H99 strains harbored variable abilities to produce titan cells compared to H99O (grey bar). KN99α in vivo has similar results than H99O in vivo. (B) Sgf29Δ and lmp1Δ deletion mutants show a decrease in titan cells generation in various H99 backgrounds in vivo compared to H99O. The complementation of the genes with the corresponding mutant rescued the phenotype observed for the parental strain in vivo. (TIFF) [file ppat.1006982.s009.tiff]

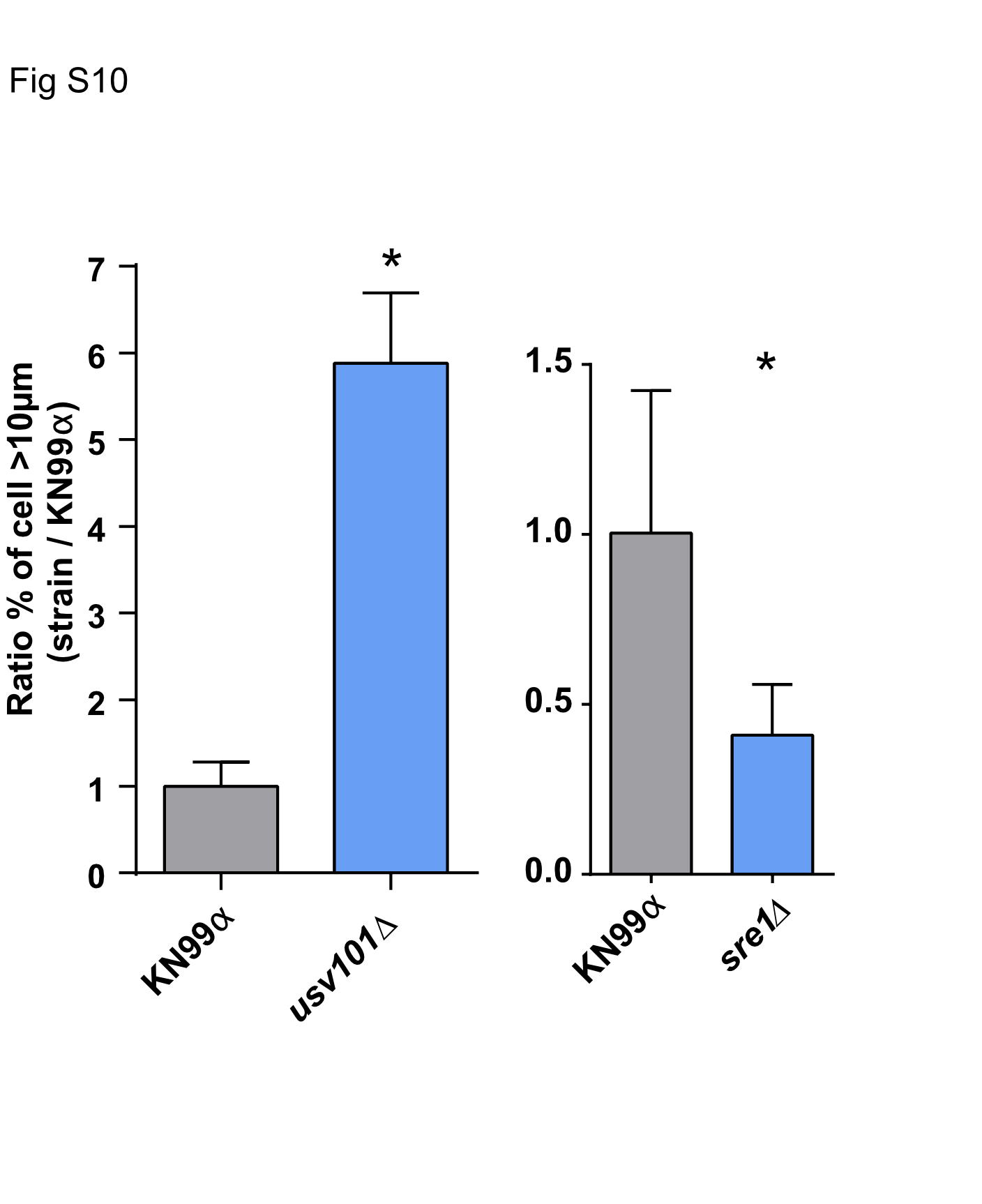

Supplement: S10 Fig — (A) usv101Δ is a repressor of titan cells formation. (B) The sre1Δ mutant strain decreased titan cells formation compared to the parental strain KN99α. The ratio to KN99α, used as a calibrator in each experiment, was calculated for each strain and results expressed as mean ±SD. To compare the experimental conditions to KN99α, Khi2 analysis was performed (*p<0.0001). (TIFF) [file ppat.1006982.s010.tiff]

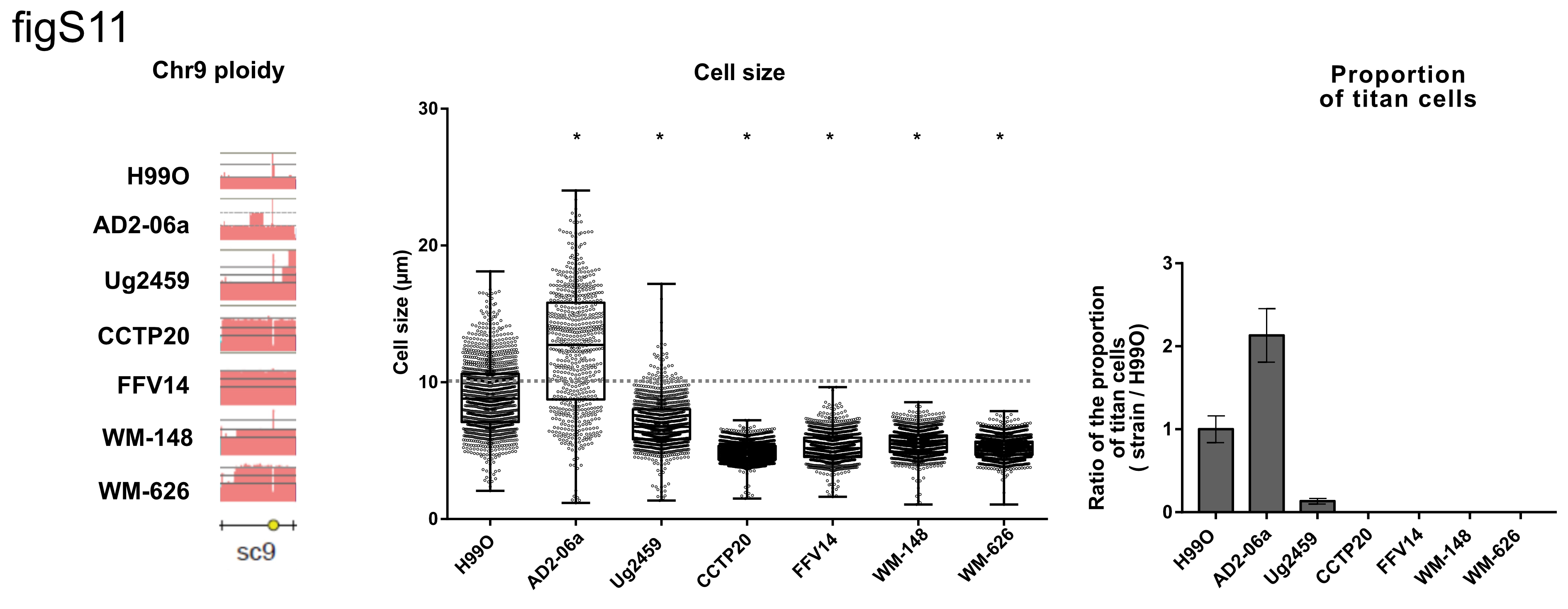

Supplement: S11 Fig — A panel of 7 clinical isolates with partial Chromosome 9 duplication (left panel) was tested for its ability to generate titan cells. Only H99O and AD2-06a exhibited increased cell sizes (middle panel). The proportion of titan cells was 67.9% (431/667) for AD2-06a, 32.1% (429/1339) for H99O, and 4.2% (51/1227), for Ug2459 (Khi2 compared to H99O, *p<0.0001) with the ratio of the proportion of titan cells to that produced in H99O shown in the right panel. (TIFF) [file ppat.1006982.s011.tiff]

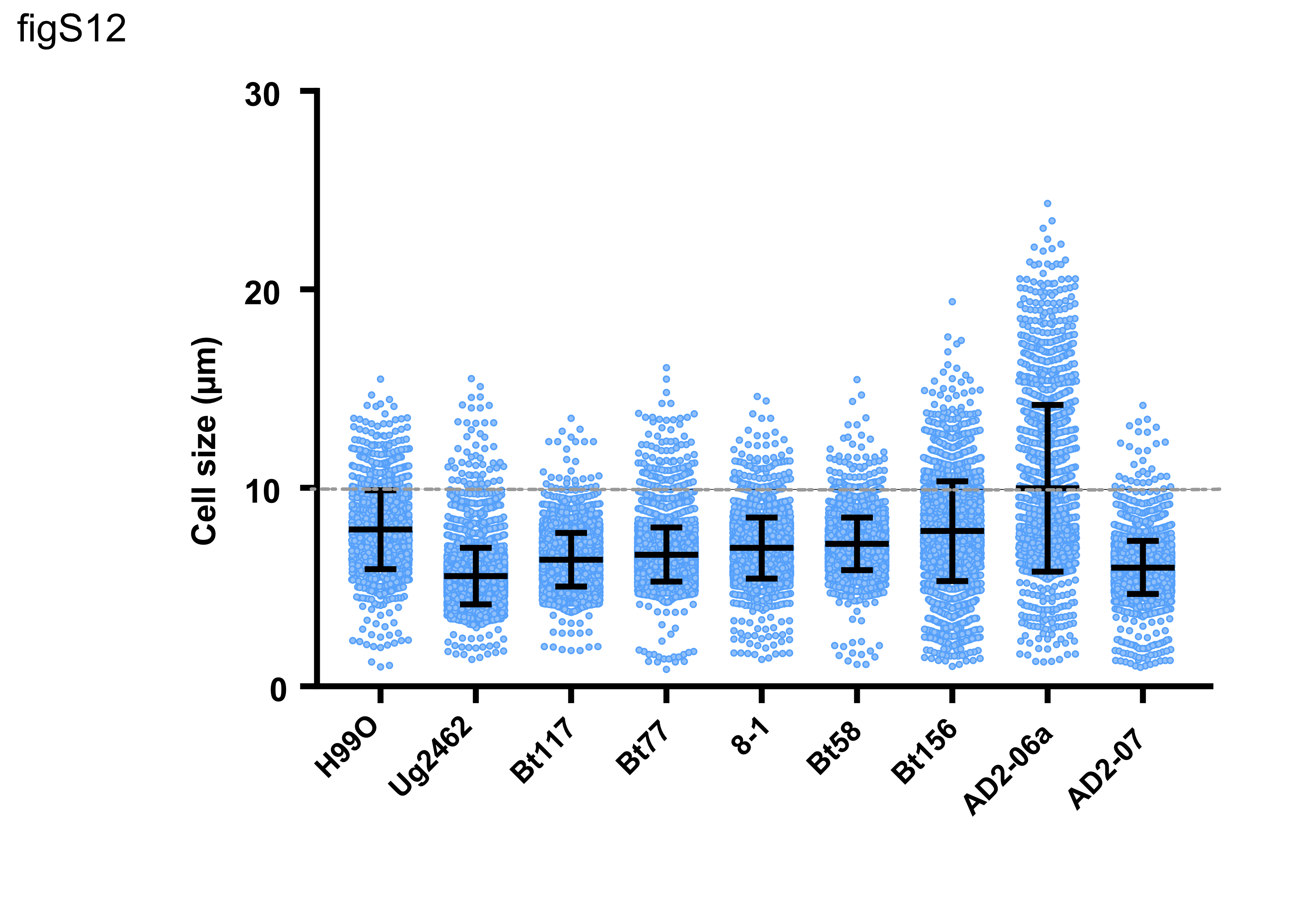

Supplement: S12 Fig — Strains with Pkr1 loss of function mutation showed a variable ability to produce titan cells depending on the resulting truncated proteins. The clinical isolate AD2-07 which did not harbor the PKR1 mutation was recovered from the CSF of an HIV-positive patient on d 13 of amphotericin B treatment while AD2-06a was recovered from its initial CSF. The median cell size (5.9 μm [5.2–6.6]) was significantly decreased in AD2-07 and increased in AD2-06a (8.5 μm [7.0–13.0]) compared to H99O (7.7 μm [6.6–9.2]) (p<0.0001). Except Bt156 (median of 7.7 μm [6.4–9.4]), the others strains had a significantly decreased median size compared to H99O (p<0.0001), 6.9 μm [6.0–7.8] for 8–1 strain; 6.5 μm [5.8–7.1] for Bt77, 6.4 μm [5.7–7.2] for Bt117; and 5.4 μm [4.7–6.1] for Ug2462. Experiments were done in triplicate and pooled. (TIFF) [file ppat.1006982.s012.tiff]

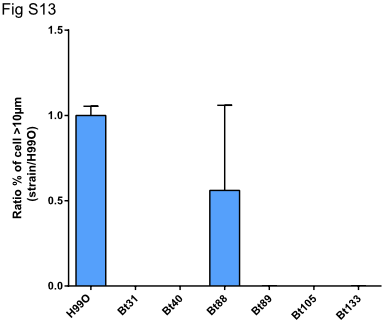

Supplement: S13 Fig — Bt88 harbored a truncated Usv101 protein due to a frameshift mutation. The titan cells generation is negative in Bt31, Bt40, Bt89, Bt105 and Bt133 and increased for Bt88 with a ratio at 0.6±0.4 and a proportion of titan cells of 21.6% (423/1958) compared to H99O 38.5% (729/1890). Experiments were done in triplicate and pooled. (TIFF) [file ppat.1006982.s013.tiff]
