# Supplementary material for: Titan cells formation in Cryptococcus neoformans is finely tuned by environmental conditions and modulated by positive and negative genetic regulators
Source: PLoS Pathog. 2018 May 18;14(5):e1006982. doi: 10.1371/journal.ppat.1006982 (PMC5959062; doi:10.1371/journal.ppat.1006982)
Supplement: S1 Table — (DOCX) [file ppat.1006982.s017.docx]

**S1 Table : Gene disrupted in AD2-06a but not in closely related isolated AD3-55a or AD3-41a**

| **Genes** | **Mutation** | **Function** |
| --- | --- | --- |
| CNAG_00039 | NSY | Uncharacterized protein |
| **CNAG_00570** | **NSY** | **cAMP-dependent protein kinase regulatory subunit** |
| CNAG_00844 | NSY | Uncharacterized protein |
| CNAG_00898 | NSY | Multidrug efflux pump |
| CNAG_01858 | NSY | Uncharacterized protein |
| CNAG_02027 | NSY | Uncharacterized protein |
| CNAG_03211 | NSY | Uncharacterized protein |
| CNAG_03319 | NSY | Phospholipid binding protein |
| CNAG_03597 | NSY | Uncharacterized protein |
| CNAG_03696 | NSY | Uncharacterized protein |
| CNAG_04602 | NSY | Uncharacterized protein |
| CNAG_05334 | NSY | Uncharacterized protein |
| CNAG_05335 | NSY | Uncharacterized protein |
| CNAG_05432 | NSY | Uncharacterized protein |
| CNAG_06698 | NSY | Phosphatase |
| CNAG_07475 | NSY | Uncharacterized protein |
| CNAG_00714 | STOP | Uncharacterized protein |
| CNAG_06796 | STOP | Uncharacterized protein |
| CNAG_05590 | STOP | Uncharacterized protein |

**NSY= non-synonymous mutation, STOP= codon STOP**
