# Supplementary material for: Titan cells formation in Cryptococcus neoformans is finely tuned by environmental conditions and modulated by positive and negative genetic regulators
Source: PLoS Pathog. 2018 May 18;14(5):e1006982. doi: 10.1371/journal.ppat.1006982 (PMC5959062; doi:10.1371/journal.ppat.1006982)
Supplement: S2 Table — (DOCX) [file ppat.1006982.s018.docx]

**S2 Table: Clinical isolates with chromosome 9 ploidy variation**

| **Strain** | **TC ratio**  **compared to H99O** | **Chromosome**  **number** | **Region** | **Copy number variation Levels** |
| --- | --- | --- | --- | --- |
| AD2-6a | 2.78 | 9 | 465 kb - 665 kb  (CNAG_04278-04359) | Diploid |
| AD4-92a | 0 | 12 | 0 - 250 kb | Diploid |
|  |  | 13 | 0 - 90 kb | Diploid |
| AD3-83a | 0 | 1 | 1880 - 2045 kb | Diploid |
| AD3-95a | 0.23 | 1 | 1785 - 1965 kb | Diploid |
| AD4-76a | 0 | 5 | 1720 - 1765 kb | Diploid |
|  |  | 11 | 190 - 310 kb | Diploid |
|  |  | 12 | 160 - 250 kb | Diploid |
