# Supplementary material for: Titan cells formation in Cryptococcus neoformans is finely tuned by environmental conditions and modulated by positive and negative genetic regulators
Source: PLoS Pathog. 2018 May 18;14(5):e1006982. doi: 10.1371/journal.ppat.1006982 (PMC5959062; doi:10.1371/journal.ppat.1006982)
Supplement: S3 Table — (DOCX) [file ppat.1006982.s019.docx]

**S3 Table: Strains harboring PKR1/CAC1/USV101 loss-of-function mutations used in this study**

| **Strain** | **Group** | **Mating type** | **Country of origin** | **Isolation source** | **Broad Project** | **SRA Accession** | **Strain description** |
| --- | --- | --- | --- | --- | --- | --- | --- |
| **8-1** | VNII | α | USA | Clinical | G27153 | SRX273085 | Litvintseva et al 2011 PLoS One, Litvintseva et al 2006 Genetics |
| **Ug2462** | VNII | α | Uganda | Clinical | G27157 | SRX273266 | Litvintseva et al 2006 Genetics |
| **Bt117** | VNI | α | Botswana | CSF /HIV+ | G26148 | SRX256502 | Litvintseva et al 2003 Euk. Cell |
| **Bt156** | VNI | α | Botswana | CSF /HIV+ | G26150 | SRX255984 | Litvintseva et al 2003 Euk. Cell |
| **Bt58** | VNI | α | Botswana | CSF /HIV+ | G26059 | SRX256524, SRX256529 | Litvintseva et al 2003 Euk. Cell |
| **Bt77** | VNI | α | Botswana | CSF /HIV+ | G26038 | SRX256435, SRX256534 | Litvintseva et al 2003 Euk. Cell |
| **AD2-06a** | VNI | α | France | Clinical | G27143 | SRX273092, SRX273094, SRX273093 | Dromer et al 2007 PLoS Med |
| **Bt31** | VNBII | α | Botswana | CSF /HIV+ | G25968 | SRX255961, SRX255966, SRX256066 | Litvintseva et al 2003 Euk. Cell |
| **Bt40** | VNBII | α | Botswana | CSF /HIV+ | G25969 | SRX256029, SRX255946 | Litvintseva et al 2003 Euk. Cell |
| **Bt88** | VNBII | a | Botswana | CSF /HIV+ | G25995 | SRX256010, SRX272109, SRX256352 | Litvintseva et al 2003 Euk. Cell |
| **Bt89** | VNBII | α | Botswana | CSF /HIV+ | G25966 | SRX255594, SRX255662 | Litvintseva et al 2003 Euk. Cell |
| **Bt105** | VNBII | α | Botswana | CSF /HIV+ | G27130 | SRX273121, SRX273122, SRX404420 | Litvintseva et al 2003 Euk. Cell |
| **Bt133** | VNBII | a | Botswana | CSF /HIV+ | G27162 | SRX273143, SRX273141, SRX273142 | Litvintseva et al 2003 Euk. Cell |
