# Supplementary material for: Titan cells formation in Cryptococcus neoformans is finely tuned by environmental conditions and modulated by positive and negative genetic regulators
Source: PLoS Pathog. 2018 May 18;14(5):e1006982. doi: 10.1371/journal.ppat.1006982 (PMC5959062; doi:10.1371/journal.ppat.1006982)
Supplement: S4 Table — (DOCX) [file ppat.1006982.s020.docx]

**S1 Table : Strains used in this study**

| Strain denomination | Reference or Source |
| --- | --- |
| H99O | J. Heitman (Duke University, Durham, NC, USA), Kirsten Nielsen, Arturo Casadevall, James Fraser |
| KN99α | Kirsten Nielsen, Madhani collection |
| H99W | Janbon *et al.,* plos genetics 2014, Kirsten Nielsen |
| H99S | Janbon *et al.,* plos genetics 2014, Kirsten Nielsen |
| H99L | Janbon *et al.,* plos genetics 2014, Kirsten Nielsen |
| H99CMO18 | Homer *et al.*, cell host microbe, 2015, Kirsten Nielsen, Madhani collection |
| *gpr4/5Δ* in H99 | Kirsten Nielsen |
| *gpr4/5Δ* in KN99α | Kirsten Nielsen |
| *sgf29Δ:SGF29* in H99S | James Fraser |
| *rim101Δ* in H99 | Kirsten Nielsen |
| *rim101Δ:RIM101* in H99 | Kirsten Nielsen |
| *sgf29Δ* in H99O | Janbon *et al.,* plos genetics 2014 |
| *sgf29Δ* in H99S | Janbon *et al.,* plos genetics 2014 |
| *sgf29Δ:SGF29* in H99S | Janbon *et al.,* plos genetics 2014 |
| *lmp1Δ* in H99S | Janbon *et al.,* plos genetics 2014 |
| *lmp1Δ:LMP1* in H99S | Janbon *et al.,* plos genetics 2014 |
| *gpr4Δ* in KN99α | Madhani collection |
| *gpr5Δ* in KN99α | Madhani collection |
| *rim101Δ* in KN99α | Madhani collection |
| *qsp1Δ* in KN99α | Madhani collection |
| *pqp1Δ* in KN99α | Madhani collection |
| *opt1Δ* in KN99α | Madhani collection |
| *tsp2Δ-1* in KN99α | Madhani collection |
| *pkr1Δ* in KN99α | Madhani collection |
| *sre1Δ* in KN99α | Madhani collection |
| *usv101Δ* in KN99α | Madhani collection |
| *pkr1Δ-1* in H99 | James Kronstad |
| *pkr1Δ-2* in H99 | James Kronstad |
| *pkr1Δ-1:PKR1* | James Kronstad |
| *pkr1Δ-2:PKR1* | James Kronstad |
| P_GAL7_::PKA1 | Choi *et al.*, molecular microbiology, 2012 |
| P_GAL7_::PKR1 | Choi *et al.*, molecular microbiology, 2012 |
| *tsp2Δ-2* | Guilhem Janbon |
| *tsp2Δ-3* | Guilhem Janbon |
| *tsp2Δ-1:TSP2-1* | Kirsten Nielsen |
| *tsp2Δ-1:TSP2-2* | Kirsten Nielsen |
| AD2-06a*:PKR1* | Kirsten Nielsen |
| AD2-06a, AD2-07, AD2-02a, AD4-37a, AD1-95a, AD4-43a, AD4-92a, AD2-79a, AD3-52a, AD5-66a, AD2-64a, AD2-82a, AD2-45a, AD4-76a, AD5-67a, AD5-45a, AD5-39a, AD1-07a, AD1-42a, AD1-79a, AD2-10a, AD2-24a, AD2-43a, AD2-47a, AD2-51a, AD2-60a, AD2-89a, AD3-20a, AD3-42a, AD3-55a, AD3-58a, AD3-63a, AD4-07a, AD4-08a, AD4-10a, AD4-39a, AD4-44a, AD4-98a, AD5-60a, AD5-64a, AD2-99a, AD3-83a, AD3-09a, AD3-11a, AD3-41a, AD3-95a, AD4-63a, AD5-53a, AD6-54a, AD6-55a, AD1-68a, AD1-83a, AD1-86a, AD1-90a, AD2-04a, AD4-47a, AD7-77 | CryptoA/D study, Dromer, PlosMedicine, 2007 |
| CCTP20, FFV14, Ug2459, WM-626, WM-148, 8-1, Bt31, Bt40, Bt58, Bt77, Bt88, Bt89, Bt105, Bt133, Bt156, Bt117, Ug2462 | Rhodes *et al*., genetics 2017; Desjardins et al., Genome research 2017 |
